# Case report: Microsurgical resection of a giant triple dumbbell shaped jugular foramen Schwannoma via retrosigmoid and transcervical approach

**DOI:** 10.3389/fonc.2024.1432835

**Published:** 2024-10-23

**Authors:** Haiying Sun, Yujuan Hu, Yun Zhu, Juanjuan Hu, Jie Yuan, Zuhong He, Huamao Cheng

**Affiliations:** ^1^ Department of Otorhinolaryngology, Union Hospital, Tongji Medical College, Huazhong University of Science and Technology, Wuhan, China; ^2^ Department of Otorhinolaryngology-Head and Neck Surgery, Zhongnan Hospital of Wuhan University, Wuhan, China

**Keywords:** jugular foramen, Schwannoma, microsurgery, transmastoid, triple dumbbell-shape

## Abstract

The surgical management of extensive jugular foramen schwannomas presents a formidable challenge, aiming for gross total resection while minimizing complications. Here, we present a case with giant triple dumbbell-shaped jugular Foramen Schwannoma. A 45-year-old male with a one-year history of a left neck mass underwent surgery. Initial misdiagnosis of submandibular gland inflammation led to persistent symptoms despite anti-inflammatory treatment. Imaging revealed a large lesion in the left cerebellar peduncle-neck-jugular foramen region (39.6 x 26.2 x 90 mm). The combination of retrosigmoid and transcervical approach was selected. Sufficient drilling of the infralabyrinthine, retrofacial area of the mastoid with facial nerve transposition is important for the safe gross total removal of the tumor. The patient underwent a gross total removal of the tumor. Facial nerve function was preserved. Although dysphagia and hoarseness complicated postoperatively, he became able to take foods orally after the surgery. In conclusion, this case underscores the successful surgical approach for a large jugular foramen Schwannoma, emphasizing the importance of precise techniques to achieve complete tumor resection while minimizing postoperative complications.

## Introduction

Jugular Foramen Schwannomas (JFSs) is a rare and challenging condition characterized by the presence of schwannomas in the jugular foramen, an anatomically complex region of the skull (1, 2). The jugular foramen region encompasses critical neurovascular structures, including the lower cranial nerves and jugular vein (3). JFSs constitute approximately 2.9–4% of all intracranial schwannomas (4). Despite its low incidence, the surgical management of extensive JFSs remains a formidable challenge due to the intricate anatomy and potential for complications. JFSs are classified based on their shape and extension. Samii’s classification categorizes JFSs into three subtypes based on tumor location and extensions: type A (tumors mainly in the cerebellopontine angle with jugular foramen enlargement), type B (tumors primarily in the foramen with intracranial extension), and type C (mainly extracranial tumors extending into the jugular foramen, forming dumbbell-shaped tumors across intracranial, jugular foramen, and extracranial compartments) (5, 6).

Attaining total resection of tumors in this area necessitates an optimal neurosurgical approach. Several approaches, such as the far lateral approach, juxtacondylar approach, and postauricular transtemporal approach, have been introduced in alignment with this classification (7). For extensive dumbbell-shaped JFS, a two-piece lateral suboccipital approach emerges as a suitable option (8). The surgical approach to these tumors is critical for achieving gross total resection while preserving vital structures and minimizing postoperative complications. Common complications associated with these tumors include facial nerve paresis, hearing disturbances, dysphagia, hoarseness, and cerebrospinal fluid (CSF) leakage, emphasizing the need for precise surgical techniques (9).

Within this context, we present the case of a 45-year-old male diagnosed with a rare giant triple dumbbell-shaped jugular foramen Schwannoma. The patient’s clinical history, initial misdiagnosis, diagnostic imaging, and the selected surgical approach bring attention to the intricacies and considerations inherent in managing such uncommon tumors. This case not only enhances our understanding of the diverse presentations of jugular foramen Schwannomas but also underscores the crucial role of personalized surgical strategies. The favorable outcome, marked by preserved facial nerve function and improved postoperative conditions, highlights the critical importance of detailed surgical planning and precise execution to achieve optimal results in patients grappling with this challenging condition.

## Case presentation

### Clinical history

**Figure 1:**
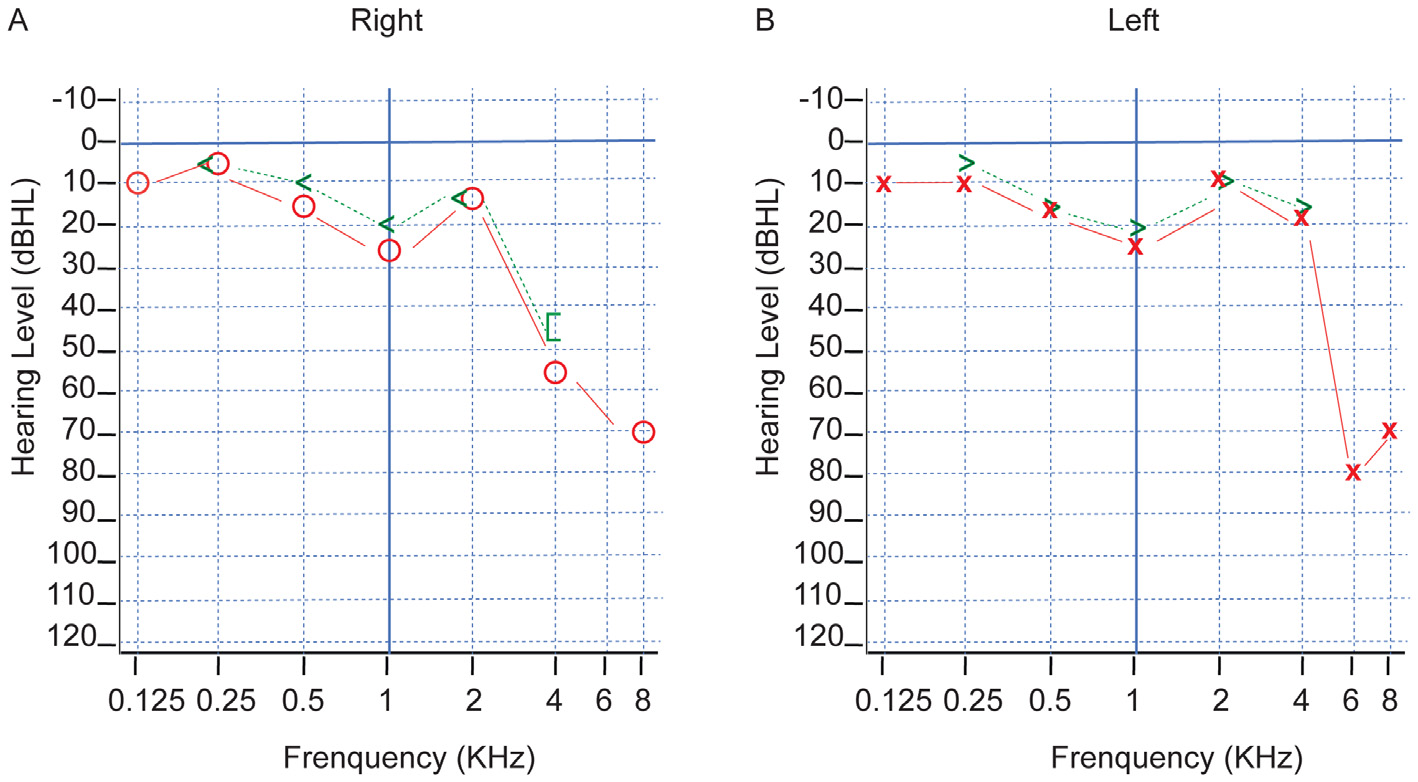
**(A, B)** Pure tone audiometry results. Preoperative hearing audiogram shows no difference in hearing between the left and right ears. Both ears demonstrate severe high frequency sensorineural hearing loss. Symbols: ○, right ear air conduction; <, left ear bone conduction; X, left ear air conduction; >, left ear bone conduction. dBhl, decibels Hearing Level; KHZ, kilohertz.

This case report has been prepared in accordance with the CARE (Case Reports) guidelines, as available on the EQUATOR Network (https://www.equator-network.org/). A 45-years-old male presents to Wuhan Union hospital complaining of a mess on the left side of the neck over the past year. A neck ultrasound conducted at a local hospital one year ago revealed a 5*4*2 cm mass on the left side of the neck. No specific treatment was administered at that time. About half year later, a repeat neck ultrasound indicated no significant change in the size of the mess. A subsequent biopsy suggested the possibility of submandibular gland inflammation. The patient received anti-inflammatory treatment, but the response was unsatisfactory. Over the past three months, the patient has experienced dizziness without presenting symptoms such as hoarseness, hearing loss, facial paralysis, or difficulty swallowing. Seeking further evaluation and treatment, the patient visited our outpatient clinic. A new audiological assessment was performed and revealed no difference in hearing between the left and right ears (**Figures 1A, B**).

### Imaging

#### Computed tomography imaging

**Figure 2:**
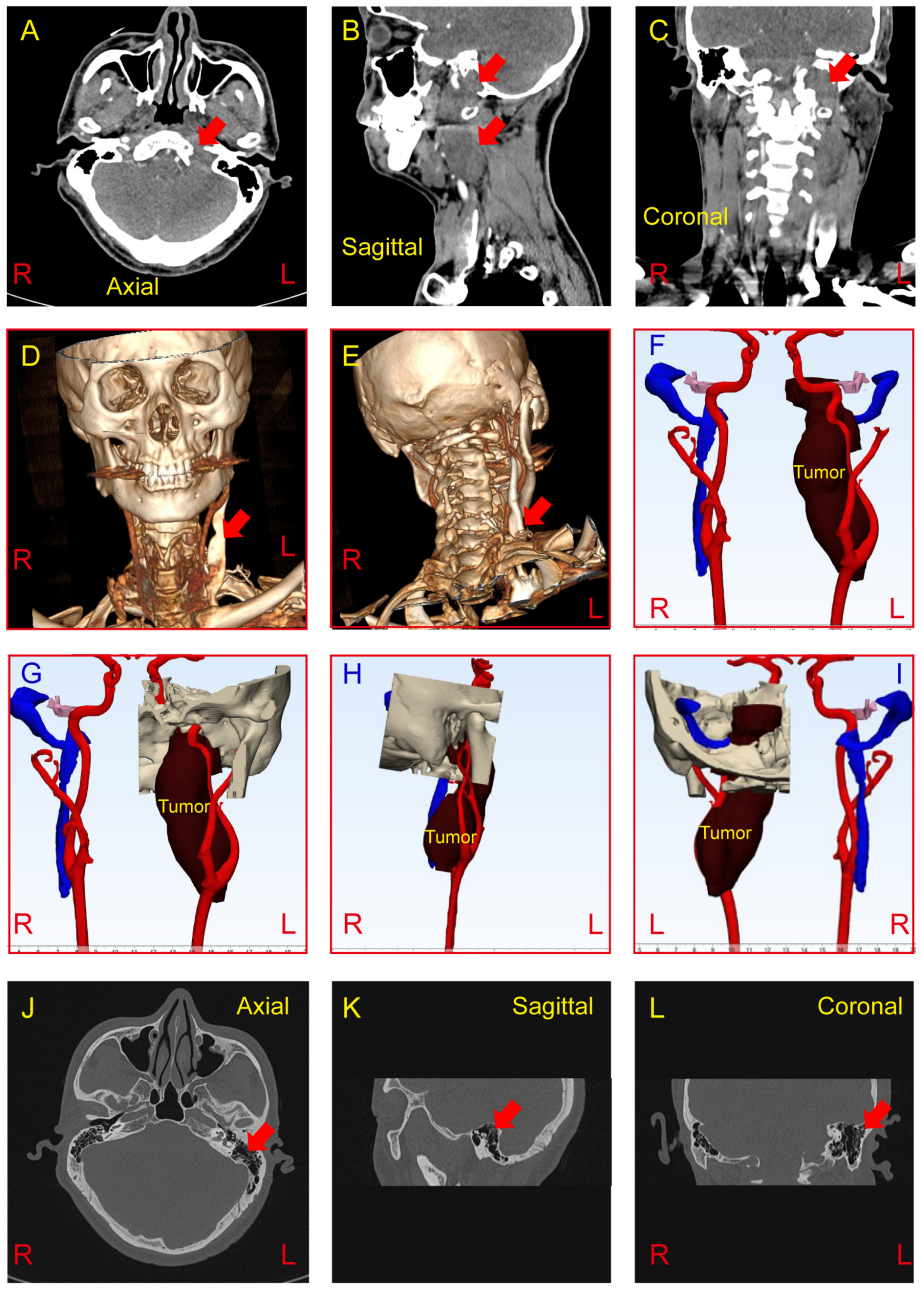
**(A-C)** CT imaging reveals an abnormal density mass in the left cerebellopontine angle, jugular foramen, and retrostyloid space, demonstrating mild to moderate heterogeneous enhancement (Red arrow). The tumor measures approximately 37*20*87mm. **(D-I)** Three-dimensional tumor model was reconstructed based on preoperative CT scan to show the spatial relationship of the tumor, arteries, and veins. **(J-L)** CT findings demonstrate no abnormalities in the bilateral external ear, middle ear, and inner ear (Red arrow show the left mastoid). R, Right; L, Light.

In the left cerebellar peduncle-neck-internal aspect of the jugular foramen region, there is a longitudinally shaped lesion with mixed long T1 and long T2 signal, showing a clear border and extending medially to the left para-pharyngeal space. The lesion exhibits significant and uneven enhancement on contrast imaging, with compression and flattening of the surrounding jugular veins and no obvious contrast agent filling. The lesion measures approximately 39.6 x 26.2 x 90 mm (**Figures 2A-C**). Based on CT scans, Radiant DICOM viewer software (version 2024.1, Germany) was applied to reconstruct the three-dimensional model to demonstrate the spatial relationship of the tumor, arteries, head, and neck (**Figures 2D-I**). Both external ear canals are normal. No obvious abnormal high or low-density signals are observed within the middle ear cavity and mastoid air cells. The ossicles on both sides show no apparent abnormalities. Additionally, there are no apparent abnormalities detected in the inner ear and internal auditory canal on both sides (**Figures 2J-L**).

#### Magnetic resonance imaging

**Figure 3:**
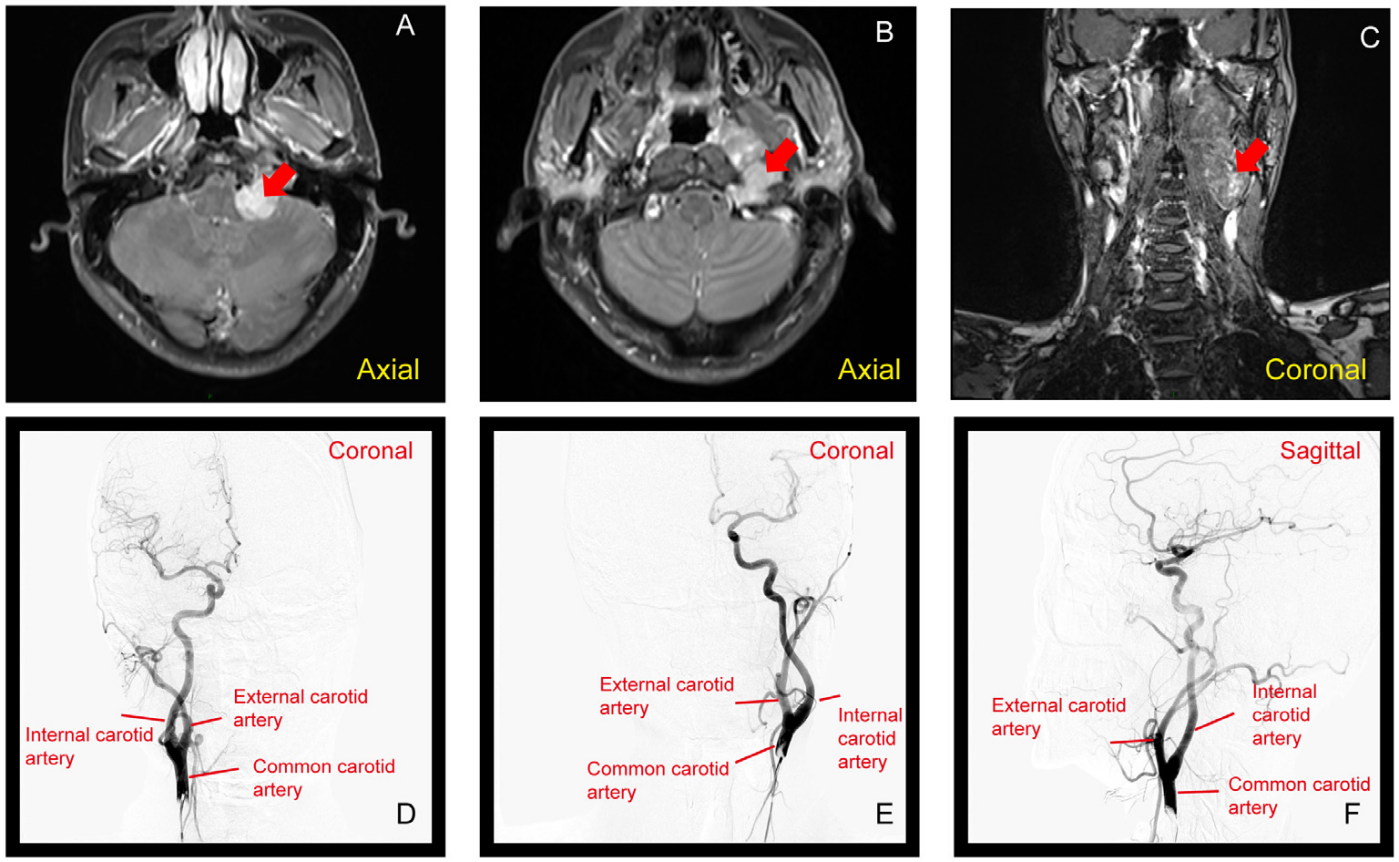
**(A–C)** MRI showing a mass at the left pontocerebellar junction to the jugular foramen, with mixed long T1 and T2 signals. The mass, measuring 40×26×90 mm, shows uneven enhancement (Red arrow show). **(D–F)** DSA with arterial injection of non-ionic iodinated contrast agent in the left and right carotid arteries, and left vertebral artery. The angiography shows no abnormal staining from the left neck to the intracranial region, with normal arterial course and morphology. The distribution of draining veins appears generally normal.

In the left pontocerebellar junction - jugular foramen, there is a mess with mixed long T1 and long T2 signals on the inner side of the neck vessel sheath. The mess has a clear border and extends inward to the left parapharyngeal space. It shows significant and uneven enhancement, measuring approximately 40*26*90mm. On the lesion plane, the left internal and external carotid arteries are displaced outward, and there is no apparent contrast agent filling in the local internal jugular vein (**Figures 3A–C**).

DSA with arterial injection of non-ionic iodinated contrast agent was performed in the left and right carotid arteries, as well as the left vertebral artery. The angiography revealed no apparent abnormal staining in the left neck to intracranial region. Arterial course and morphology were normal, and no obvious abnormalities were observed. The distribution of various draining veins showed a generally normal pattern (**Figures 3D–F**).

### Surgery

A C-shaped postauricular skin incision is made. It begins at the upper edge of the auricle, curves 4 to 5 cm behind the postauricular sulcus, and slants inferiorly to end the lower border of the sternocleidomastoid muscle (**Supplementary Figure S1A**). User Grinding away the cortical and mastoid air cells of the mastoid, exposing the jugular foramen area, excising the mass in the jugular foramen area, and separating the tumor, vessels, and nerves downward. Dissecting the tumor from the dura mater and the overlying bony plate, grinding off the posterior cranial fossa bony plate, incising the dura mater, exposing the mass, and gradually performing intracapsular excision of the mass. Noting the convergence of the posterior cranial nerves into the mass and completely peeling off the mass (**Supplementary Figures S1B-E**).

### Pathological examination

**Figure 4:**
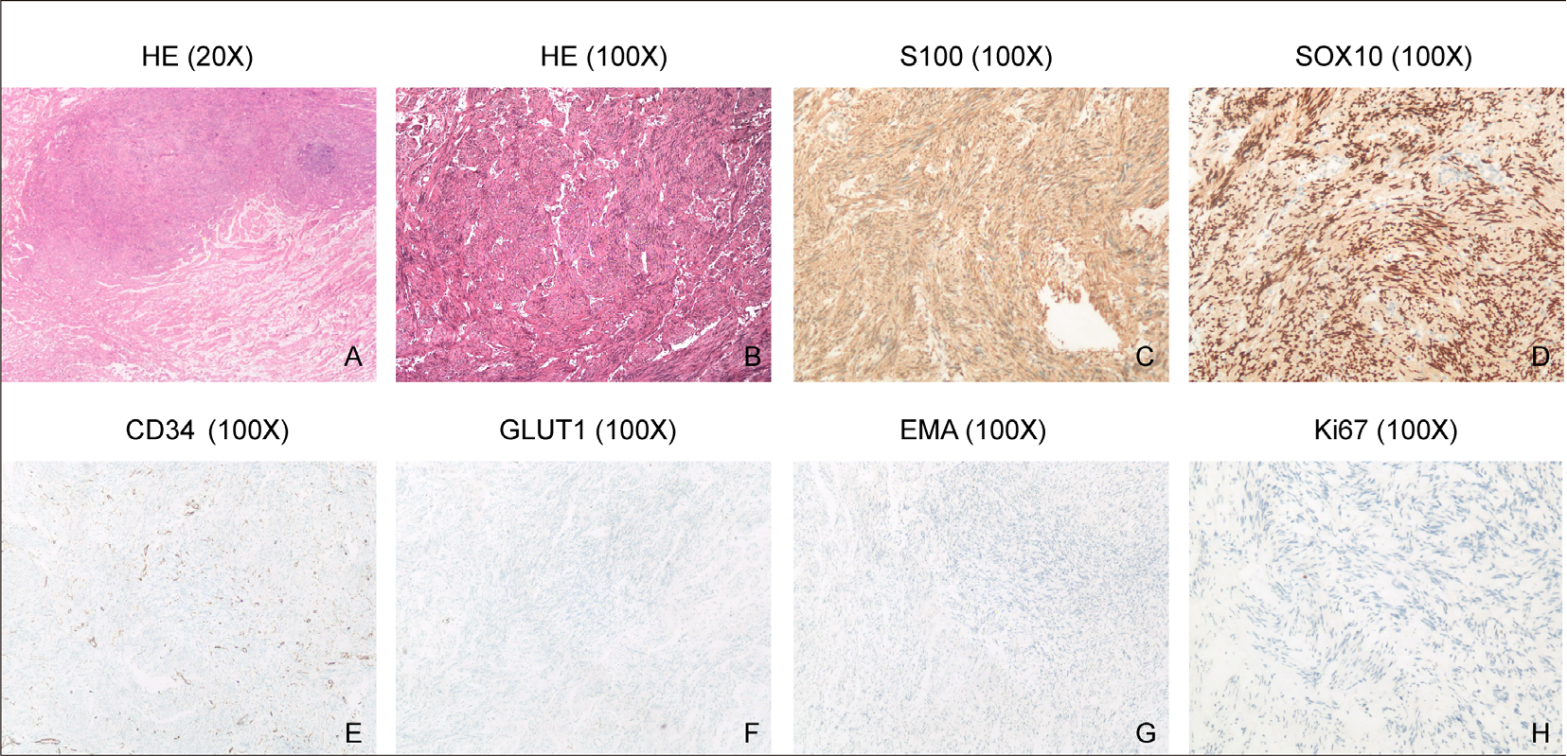
H&E staining and Immunohistochemistry. **(A, B)**, HE staining shows spindle-shaped schwannoma cells. **(C-G)**, Immunohistochemical analysis demonstrates positive staining for S100, SOX10 and CD34 in the tumor, but negative staining for GLUT1 and EMA. **(H)**, Ki67 immunostaining reveals a low proliferation index of 1% in the tumor, indicating a relatively low rate of cell proliferation.

The conclusive histopathologic examination definitively established the diagnosis of Schwannoma, as depicted in **Figures 4A, B**. The immunohistochemical analysis of the tumor further substantiated the diagnosis, demonstrating positive staining for S100, SOX10, and CD34 (**Figures 4C–E**). Notably, the tumor exhibited negative immunoreactivity for GLUT1 and EMA (**Figures 4F, G**). Additionally, the proliferation index, as indicated by Ki67, was notably low at 1% (**Figure 4H**), providing evidence of the tumor’s limited proliferative activity. These findings collectively support the accurate identification of the lesion as Schwannoma and provide a comprehensive immunohistochemical profile for further characterization.

### Post-surgery magnetic resonance imaging

The patient underwent a follow-up MRI seven months post-surgery, which revealed the following: the soft tissue on the left side of the neck was thinner compared to the right side (**Supplementary Figures S2A–C**). A mixed signal was observed in the left jugular foramen area, with a small amount of unevenly enhanced solid components (**Supplementary Figures S2A–F**). However, no evidence of tumor recurrence was detected.

## Discussion

The presented case of a giant triple dumbbell-shaped jugular foramen Schwannoma underscores the intricate nature of surgical management and the importance of tailored strategies for optimal outcomes. Jugular Foramen Schwannomas (JFSs) are rare, constituting approximately 2.9–4% of all intracranial schwannomas, and pose significant challenges due to their location and potential complications (7).

Exiting literature on JFSs, including studies such as those by Takahashi M et al., Bakar B. et al., and others (10–13), provides valuable insights into the classification, surgical management, and outcomes of these tumors. Samii’s classification categorizes JFSs into three subtypes (type A, B, and C) based on their shape and extension (5). However, the complexity of cases within each subtype often necessitates tailored approaches. For instance, type C tumors, which involve extensive extracranial and intracranial extensions, require particularly nuanced surgical planning. The literature also highlights various surgical approaches, with retrosigmoid and transcervical routes being commonly employed based on tumor location and extent.

Despite these advances, the treatment of JFSs remains challenging, with case series often reporting a balance between achieving gross total resection and minimizing neurological deficits. Recent studies have emphasized the importance of intraoperative neuromonitoring and advanced imaging techniques in improving surgical outcomes, yet each case presents unique anatomical and pathological considerations that must be addressed individually (14, 15).

Our case falls within the type C category, involving a mainly extracranial tumor extending into the jugular foramen, forming a dumbbell-shaped tumor across intracranial, jugular foramen, and extracranial compartments (4, 14). The tumor,s extensive involvement required a combination of retrosigmoid and transcervical approaches, with a key innovation being the preservation of the facial nerve without transposition. This approach, not commonly highlighted in the literature, was critical in maintaining facial nerve function, as evidenced by the patient’s postoperative recovery.

Furthermore, the precise drilling technique employed in the infralabyrinthine, retrofacial area of the mastoid, without compromising the facial nerve, represents a significant surgical advancement. The use of intraoperative neuromonitoring throughout the procedure further ensured the safety of the patient.

Precise surgical planning and execution are paramount, given the involvement of critical neurovascular structures. The successful gross total removal of the tumor in our case, despite the initial misdiagnosis and challenges posed by the extensive nature of the Schwannoma, highlights the effectiveness of the chosen surgical approach. Notably, meticulous drilling of the infralabyrinthine, retrofacial area of the mastoid without facial nerve transposition played a crucial role in ensuring both safety and efficacy.

Postoperatively, the patient experienced complications such as dysphagia and hoarseness. However, these transient issues were outweighed by the overall success of the surgery, as the patient regained the ability to take foods orally, and facial nerve function was preserved. These outcomes underscore the importance of weighing potential complications against the benefits of tumor resection.

The pathological examination confirmed the diagnosis of Schwannoma, with immunohistochemical analysis providing valuable insights into the tumor’s characteristics. Positive staining for S100, SOX10, and CD34, coupled with low Ki67 proliferation index, aligns with typical Schwannoma profiles. The negative immunoreactivity for GLUT1 and EMA further supports the accurate identification of the lesion.

In cases of residual or recurrent tumors, stereotactic radiosurgery offers a viable adjunctive treatment. Gamma Knife radiosurgery is particularly effective for lesions extending down to the C3 vertebra. For lesions adjacent to critical neural structures, fractionated radiosurgery may be indicated to minimize damage to surrounding tissues. Additionally, the incorporation of radioenhancers can potentiate the therapeutic dose to the tumor while sparing normal tissues (16, 17). These advanced techniques broaden the scope of effective management options for complex JFSs.

In conclusion, while the literature provides a foundation for understanding the complexities of JFS management, this case contributes a unique perspective on the surgical treatment of giant triple dumbbell-shaped tumors. The innovative approach, particularly in preserving facial nerve function without transposition, alongside the meticulous pre-operative planning and execution, adds significant value to the current body of knowledge. This case not only underscores the importance of individualized surgical strategies but also highlights the potential for improved outcomes through careful application of advanced techniques and technologies in complex JFS cases.

## Data Availability

The original contributions presented in the study are included in the article/**Supplementary Material**. Further inquiries can be directed to the corresponding authors.

## Supplementary material

Supplementary Figure 1 Intraoperative images. **(A)** Surgical incision starting from behind the ear, extending posteriorly, progressing to the neck, and terminating at the inferior border of the sternocleidomastoid muscle. **(B)** Excised mass during the surgery. **(C, D)** Intraoperative visualization revealing tumor infiltration into the intracranial space with associated skull base damage. **(E, F)** Intraoperative observation highlighting the close relationship of the tumor with vascular and neural structures. IAA: internal auditory nerve; FN: Facial nerve.

Supplementary Figure 2 Postoperative enhanced MRI. **(A-C)** T1-enhanced sequences show that the soft tissues in the left neck are thinner compared to the contralateral side (Red asterisk). **(A-F)** T1-enhanced sequences also demonstrate heterogeneous signal intensity in the left jugular foramen area, demonstrating slight uneven enhancement with a solid component (Red arrow head).

Supplementary Video 1 The external auditory canal is closed to prevent any potential complications. The cortical and mastoid air cells are ground away, revealing the jugular foramen area. The mass in the jugular foramen is excised, with the tumor, vessels, and nerves dissected downward. The tumor is carefully separated from the dura mater and underlying bone, and the posterior cranial fossa bony plate is removed. Following the incision of the dura mater, the mass is exposed and an intracapsular excision is performed. The posterior cranial nerves converging into the mass are identified and the mass is fully excised.
