# Supplementary figures and images for: Case report: Microsurgical resection of a giant triple dumbbell shaped jugular foramen Schwannoma via retrosigmoid and transcervical approach
Source: Front Oncol. 2024 Oct 23;14:1432835. doi: 10.3389/fonc.2024.1432835 (PMC11538005; doi:10.3389/fonc.2024.1432835)

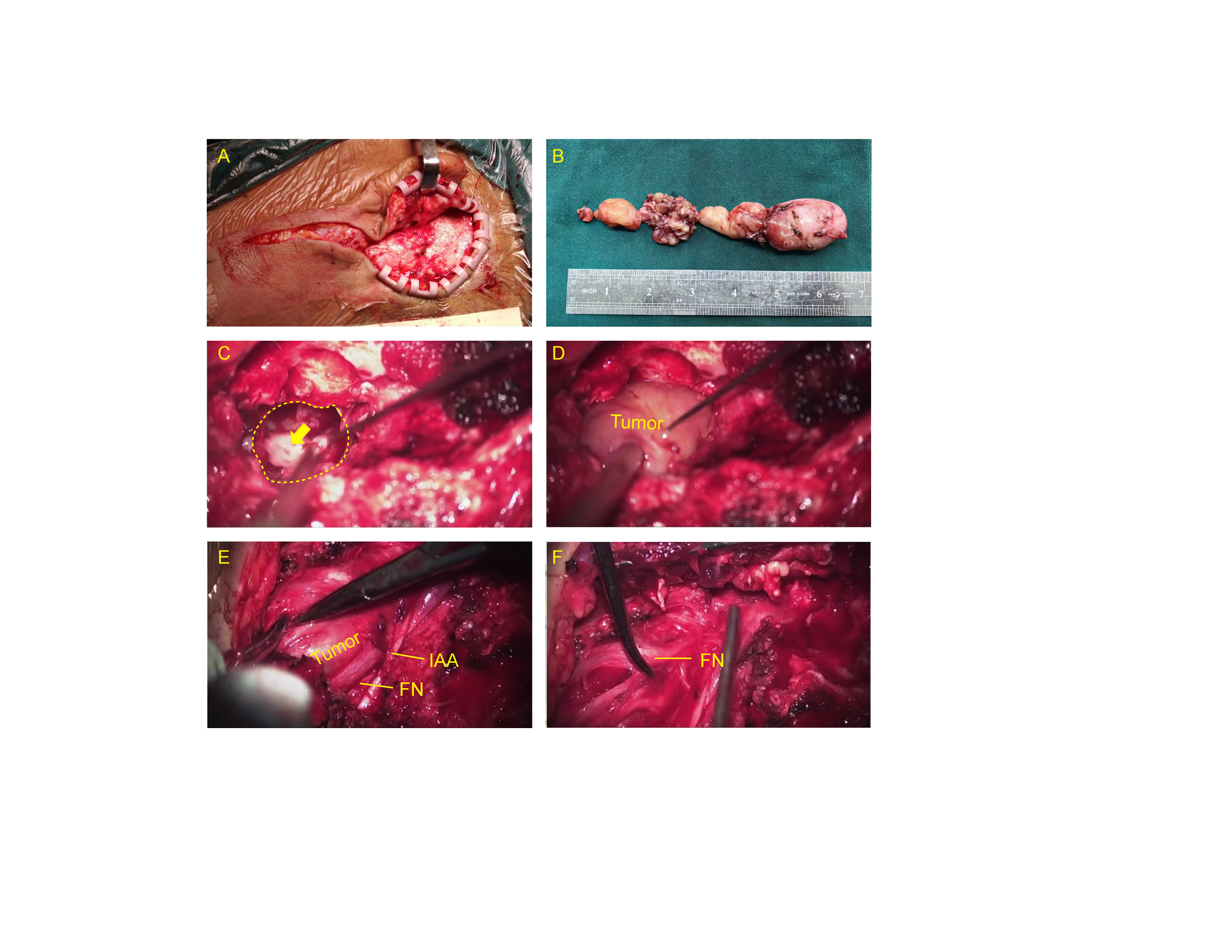

Supplement: Supplementary Figure 1 — Intraoperative images. (A) Surgical incision starting from behind the ear, extending posteriorly, progressing to the neck, and terminating at the inferior border of the sternocleidomastoid muscle. (B) Excised mass during the surgery. (C, D) Intraoperative visualization revealing tumor infiltration into the intracranial space with associated skull base damage. (E, F) Intraoperative observation highlighting the close relationship of the tumor with vascular and neural structures. IAA: internal auditory nerve; FN: Facial nerve. [file Image1.tif]

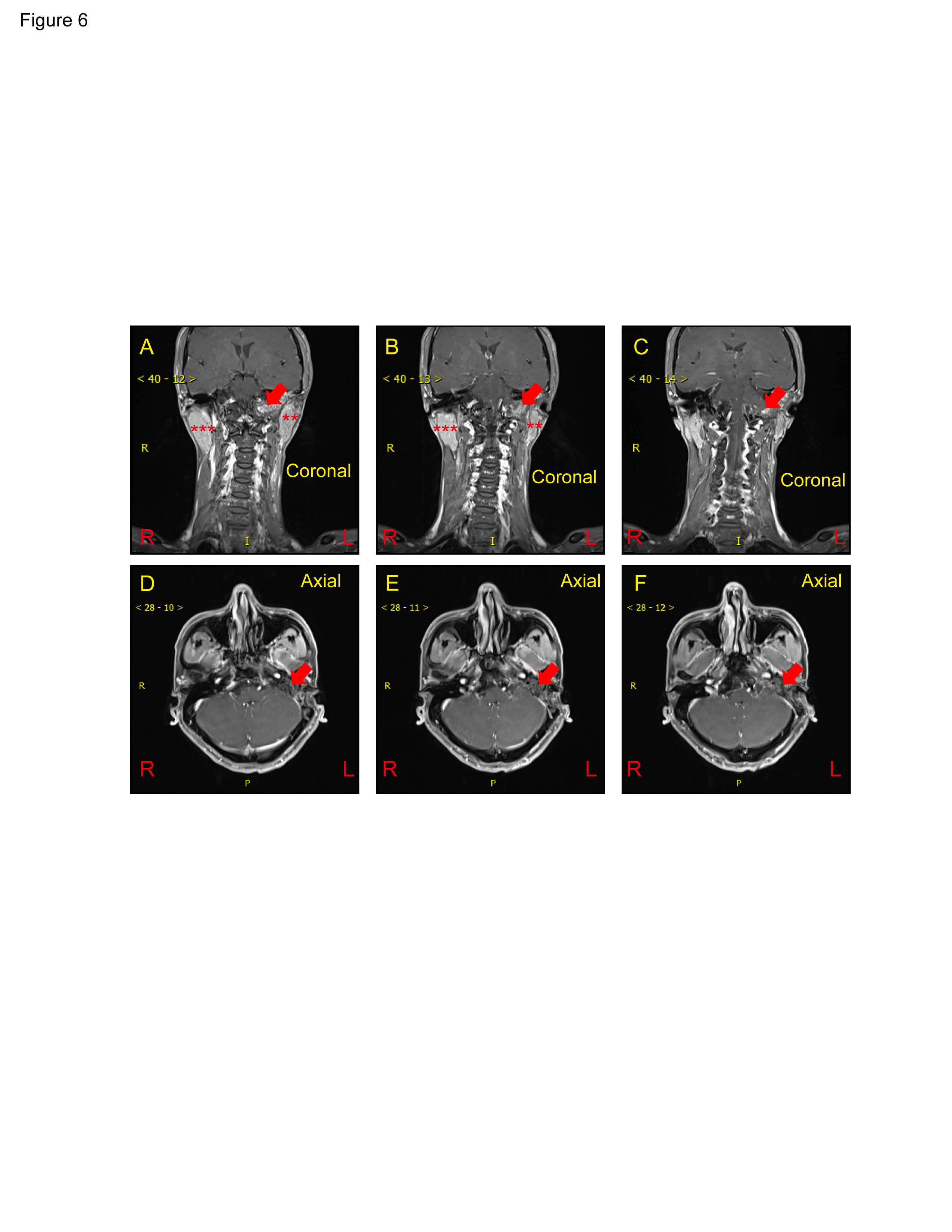

Supplement: Supplementary Figure 2 — Postoperative enhanced MRI. (A-C) T1-enhanced sequences show that the soft tissues in the left neck are thinner compared to the contralateral side (Red asterisk). (A-F) T1-enhanced sequences also demonstrate heterogeneous signal intensity in the left jugular foramen area, demonstrating slight uneven enhancement with a solid component (Red arrow head). [file Image2.tif]
